# Inhibition of Galectin-9 sensitizes tumors to anthracycline treatment via inducing antitumor immunity

**DOI:** 10.7150/ijbs.84108

**Published:** 2023-08-28

**Authors:** Xian Sun, Wei-Jan Wang, Jilu Lang, Riyao Yang, Wan-Jou Shen, Linlin Sun, Jung-Mao Hsu, Li-Chuan Chan, Chia-Wei Li, Weiya Xia, Baozhen Ke, Guodong Yao, Kebin Huang, Pei-Chih Lee, Paul B. Koller, Mien-Chie Hung

**Affiliations:** 1 Department of Oncology, The Seventh Affiliated Hospital, Sun Yat-Sen University, Shenzhen, P. R. China.; 2 Departments of Molecular and Cellular Oncology, The University of Texas MD Anderson Cancer Center, Houston, TX 77030, USA.; 3 Department of Medical Oncology, Harbin Medical University Cancer Hospital, Harbin, P. R. China.; 4 Department of Biological Science and Technology, College of Life Sciences, China Medical University, Taichung, Taiwan.; 5 Cancer Biology and Precision Therapeutics Center and Research Center for Cancer Biology, China Medical University, Taichung, Taiwan.; 6 Department of Cardiac Vascular Center, The Seventh Affiliated Hospital, Sun Yat-Sen University, Shenzhen, P. R. China.; 7 Antibody Therapeutics, Inc., Hayward, CA 94545, USA.; 8 Graduate Institute of Biomedical Sciences, China Medical University, Taichung, Taiwan.; 9 Tianjin Key Laboratory of Lung Cancer Metastasis and Tumor Microenvironment, Lung Cancer Institute, Tianjin Medical University General Hospital, Tianjin, China.; 10 Institute of Biomedical Sciences, Academia Sinica, Taipei, Taiwan.; 11 Department of Pathology, Harbin Medical University Cancer Hospital, Harbin, P. R. China.; 12 Department of Pathology, Affiliated Hospital of Qingdao University, Harbin, P. R. China.; 13 State Key Laboratory for Chemistry and Molecular Engineering of Medicinal Resources, School of Chemistry & Pharmacy, Guangxi Normal University, Guilin, Guangxi, P. R. China.; 14 Department of Hematology and Hematopoietic Cell Transplantation, City of Hope National Medical Center, Duarte, California, USA.; 15 Institute of Biochemistry and Molecular Biology, China Medical University, Taichung, Taiwan.; 16 Molecular Medicine Center, China Medical University Hospital, China Medical University, Taichung, Taiwan.; 17 Department of Biotechnology, Asia University, Taichung, Taiwan.

**Keywords:** anthracyclines, Galectin-9, tumor microenvironment, STING, antitumor immunity

## Abstract

Anthracyclines are a class of conventionally and routinely used first-line chemotherapy drugs for cancer treatment. In addition to the direct cytotoxic effects, increasing evidence indicates that the efficacy of the drugs also depends on immunomodulatory effects with unknown mechanisms. Galectin-9 (Gal-9), a member of the β-galactoside-binding protein family, has been demonstrated to induce T-cell death and promote immunosuppression in the tumor microenvironment. Here, we asked whether anthracycline-mediated immunomodulatory activity might be related to Gal-9. We found that combining doxorubicin with anti-Gal-9 therapy significantly inhibited tumor growth and prolonged overall survival in immune-competent syngeneic mouse models. Moreover, Gal-9 expression was increased in response to doxorubicin in various human and murine cancer cell lines. Mechanistically, doxorubicin induced tumoral Gal-9 by activating the STING/interferon β pathway. Clinically, Gal-9 and p-STING levels were elevated in the tumor tissues of breast cancer patients treated with anthracyclines. Our study demonstrates Gal-9 upregulation in response to anthracyclines as a novel mechanism mediating immune escape and suggests targeting Gal-9 in combination with anthracyclines as a promising therapeutic strategy for cancer treatment.

## Introduction

Breast cancer is the most common cancer and the second leading cause of cancer-related death in women 1. In the past twenty years of early diagnosis, neoadjuvant and adjuvant chemotherapy with anthracycline has significantly reduced the mortality from breast cancer 2. Anthracyclines, such as doxorubicin and epirubicin, are commonly used in clinical treatment 3. The drug action of anthracycline targets tumor DNA through the topoisomerase II enzyme, inhibiting downstream protein expression and causing cell membrane dysfunction in cancer cells 4. Although anthracycline has improved patient survival, the increase in chemoresistance poses a clinical challenge in treating breast cancer 5.

Immunogenic cell death (ICD) is a form of cell death that elicits an immune response, contrary to "silenced" cell death, such as apoptosis. It is believed that tumor cells undergoing ICD express or release damage-associated molecular patterns (DAMPs) to activate or inhibit immune cells 6. In addition, anthracyclines, including several chemotherapy drugs, have been shown to induce ICD 7. Treatment of tumor cells with anthracycline-induced calreticulin (CRT) membrane translocation is essential for tumor cell phagocytosis by dendritic cells and the antitumor immune response 8. Furthermore, anthracyclines stimulate the production of type I interferons (IFNs) by tumor cells through activation of the endosomal pattern recognition receptor (PRR) Toll-like receptor 3 (TLR3), contributing to the efficacy of chemotherapy 9. However, IFNs also upregulate the expression of multiple immune inhibitory programs, including the PD-1 ligands PD-L1 and PD-L2 10 and the TIM-3 ligand galectin-9 11.

Galectin-9 (Gal-9) is a member of the galectin family of lectins, and Gal-9 induces cell death in T helper cells and is dependent on Tim-3 through calcium aggregation 12. In addition, a recent study demonstrated that Gal-9 induces tumor-associated macrophages and promotes immune suppression via interaction with Dectin 1 13. Moreover, Gal-9 has also been demonstrated to bind 4-1BB and promote 4-1BB aggregation to induce functional activity in immune cells 14. Hence, Gal-9 might have a broader role in immune regulation. Recently, inhibition of Gal-9 was a promising therapeutic target in treating various types of cancers. Therefore, blocking Gal-9 or inhibiting Gal-9 expression in the tumor microenvironment is important to improve immunotherapy for cancer patients 15.

Recently, accumulating studies have indicated that conventional anticancer drugs affect the efficacy of immunotherapy 16. Clarifying the crosstalk between chemotherapy drugs such as anthracycline-induced ICD and cancer immune checkpoint proteins can improve more efficient combinatorial therapy. Although the effects of anthracyclines have shown promising results in breast cancer, the detailed mechanism of anthracycline-induced Gal-9 expression is still unknown.

Here, we show that anthracyclines such as doxorubicin and epirubicin induce Gal-9 expression through the STING/IFNβ axis and that combined therapy with anti-Gal-9 produced synergistic antitumor effects. These results suggested that Gal-9 expression contributes to adaptive immune resistance that limits the immunogenicity of ICD and that, combined with anti-Gal-9, is a promising strategy for cancer treatment with immunogenic chemotherapy and radiotherapy that induce IFN-I expression in breast cancer.

## Materials and Methods

### Antibodies, chemicals, and cell lines

CT26 cells (mouse colon adenocarcinoma) were obtained from the National Cancer Institute and maintained at 37 °C and 5% CO_2_ in DMEM supplemented with 10% fetal bovine serum (FBS). All other cell lines were obtained from ATCC. The mouse mammary carcinoma cell line (EMT6) was maintained in RPMI-1640 supplemented with 10% FBS, and all other cell lines were maintained as recommended by ATCC. All cell lines were mycoplasma free. Unless specified otherwise, cells were treated with anthracyclines (Sigma) at a concentration of 1 μg/ml in media for 24 hours. Gal-9 antibody (clone OTI1G3) was purchased from Bio-Rad, and STING antibody (19851-1-AP) was purchased from Proteintech. The Gal-9 antibody for immunohistochemical staining (IHC) in mouse tissue was purchased from Bioss.

### Plasmids and transfection

CRISPR‒Cas9 knockout of Gal-9 in MB-231 cells, DNA oligos 5'-caccgGGCGATGGTAGTATTCAAAC-3' and 5'-aaacGTTTGAATACTACCATCGCCc-3' were annealed and cloned into BsmBI-digested pLentiCRISPR v2 (Addgene) under the control of the type 3 RNA polymerase III promoter U6. Lentiviruses were generated, and MB-231 cells were transduced and selected with puromycin, as described above. EMT6 and CT26 cells were transfected with a pGIPZ shRNA vector (control; Thermo Fisher Scientific, Rockford, IL, USA) or pGIPZ shRNA against STING to knockdown its expression. The STING shRNA sequences were as follows (5´ to 3´): shRNA1: 5´ AGAGGTCACCGCTCCAAATAT 3´ and shRNA2: 5´ TTGTCTCTAGCACTGGTAT 3´ (targeting the 3´-untranslated region).

For the generation of stable cancer cells using retroviral infection, recombinant retroviruses were produced by cotransfecting HEK 293T cells (Clontech) with the DVPR plasmid and VSV-G plasmids using Lipofectamine 3000 (Invitrogen). Supernatants containing viruses were harvested 48 hours after transfection, centrifuged to eliminate cell debris, and filtered through 0.22 μm filters. Cancer cells at ~70% confluency were cultured in virus-containing medium for one day to infect cells. Stable clones of different constructs were selected and maintained in culture medium with 2 μg/ml puromycin.

### Real-time PCR (RT‒PCR)

Total RNA was isolated using TRIzol reagent (Invitrogen). First-strand cDNA was prepared using the PrimeScript 1st strand cDNA Synthesis Kit (Takara) according to the manufacturer's protocol with 1 μg of total RNA. All RT-PCRs were performed in a 20-μl mixture containing 1 × SYBR Green Master Mix (Takara), 0.2 μmol/L of each primer, and 2 μl of cDNA template. We used the primers 5'-ACAGACTTACAGGTTACCTCCGA-3' and 5'-CATCTGCTGGTTGAATGCTT-3' for human INFβ; 5'-GGAGCGAGATCCCTCCAAAAT-3' and 5'-GGCTGTTGTCATACTTCTCATGG-3' for human glyceraldehyde 3-phosphate dehydrogenase (GAPDH); 5'-CCACCACAGCCTCTCCATCAAC-3' and 5'-CAAGTGGAGAGCAGTTGAGGACA-3' for mouse INFβ; and 5'-CATCACTGCCACCCAGAAGACTG-3' and 5'-ATGCCAGTGAGCTTCCCGTTCAG-3' for mouse GAPDH. Real-time PCR was performed using the Applied Biosystem 7500 system under the following cycling conditions: 95 °C for 30 s, 40 cycles of 95 °C for 5 s and 60 °C for 34 s, denaturation at 95 °C for 30 s, annealing by 40 cycles at 95 °C for 5 s, and extension at 60 °C for 34 s, followed by the melting curve stage. The relative INFβ expression level was normalized to that of GAPDH.

### Assay for Anthracycline-induced IFNβ secretion

Cells (1x10^6^) were seeded in 6-well plates. After 12 hours in culture, fresh medium with 1 µg/ml doxorubicin or epirubicin (Sigma) was added for 24 hours. The conditioned medium was then harvested and measured for IFNβ using mouse IFNβ colorimetric ELISA kit (Thermo Scientific).

### Syngeneic mouse tumor models and treatments

Four-week-old female BALB/cJ and SCID mice were purchased from The Jackson Laboratory and were allowed to acclimate to the housing facility for at least two weeks before experiments. All animal experiments were performed following The University of Texas MD Anderson Cancer Center (MDACC) Institutional Animal Care and Use Committee (IACUC) guidelines (protocol number: #00001334-RN01) in an MDACC AAALAC-accredited barrier facility vivarium.

Tumorigenicity assays were performed using mouse subcutaneous breast and liver cancer models. EMT6 and CT26 cancer cells (1×10^5^) were subcutaneously injected into the right inguinal fold regions of mice. Mice were randomized into treatment groups 7-9 days later when tumors were palpable in most mice and treated with doxorubicin (4 mg/kg) or PBS. For antibody-based drug intervention, 100 μg of anti-mouse Gal-9 monoclonal antibody (mAb) (BioXCell) or rat IgG (control; BioXCell) was injected intraperitoneally every two days four times after doxorubicin injection. Subcutaneous tumors were measured using a caliper, and orthotopic tumors were evaluated using high-frequency ultrasound (Vevo 2100 imaging system: FUJIFILM VisualSonics Inc., Toronto, Ontario, Canada). Tumor volumes were calculated using the formula (length × width^2^)/2. At the experimental endpoint, mice were killed using CO^2^ exposure followed by cervical dislocation, and tumors were excised for subsequent histological analysis or processed immediately for flow cytometric analyses.

### Flow cytometric analysis

To detect cell surface Gal-9 expression, the cells were resuspended in phosphate-buffered saline (PBS) and stained with APC anti-mouse Gal-9 antibody (BioLegend) as previously described using standard protocols for flow cytometry 17. An isotype IgG antibody was used as a negative control. Stained cells were evaluated using a BD FACSCanto II cytometer, and data were analyzed using FlowJo software. To analyze cytotoxic T lymphocyte (CTL) profiles and Gal-9 levels in mouse tumor samples, a Mouse Tumor Dissociation Kit (Miltenyi Biotec) and gentleMACS Octo Dissociator (Miltenyi Biotec) were used to digest mouse tumors into single cells. After the removal of red blood cells and hybridization with CD16/CD32 antibody (TruStain fcX, BioLegend), single cells were stained for flow cytometry according to standard protocols with antibodies against the following: PE-CD45 (BioLegend), PerCP-CD3 (BioLegend), APC/Cy7-CD8a (BioLegend), and APC-Gal-9 (BioLegend). For further intracellular staining, cells were fixed, permeabilized, and stained with Pacific Blue granzyme B (BioLegend). Stained cells were analyzed using a BD FACSCanto II cytometer (BD Biosciences). Data were analyzed by using the FlowJo software program.

### Immunohistochemical staining of human breast samples

To further validate our findings in human cancer patient samples, we analyzed the correlations between p-STING levels and Gal-9 expression in human breast tumor specimens using IHC. Briefly, tissue samples were incubated with antibodies against Gal-9 (RD System) and p-STING (Cell Signaling) and then incubated with an avidin-biotin-peroxidase complex. Visualization was performed using 3-amino-9-ethylcarbazole (AEC) chromogenic substrate. Fisher's exact test and the Spearman rank correlation coefficient were used for statistical analysis, and P values less than 0.05 were considered statistically significant. For histological scoring, the staining intensity was ranked into 1 of 3 groups: high (score 3), medium (score 2), and low (score 1 and 0).

### Statistical analysis

Unless noted otherwise, graphing and statistical analyses were performed using Prism 8 (GraphPad). Unpaired two-tailed t tests were used to compare two groups, while ordinary one-way ANOVA followed by Tukey's multiple comparison tests were used to compare multiple treatment groups. Spearman's test was used to assess the correlation between continuous variables. The area under the curve was used to compare tumor growth kinetics between treatment groups. Log-rank (Mantel‒Cox) tests were used for the comparison of survival curves. NS, not significant (P > 0.05); *P < 0.05; **P < 0.01; ***P < 0.001.

## Results

### Anti-Gal-9 therapy in combination with doxorubicin improved antitumor activity

**Figure 1:**
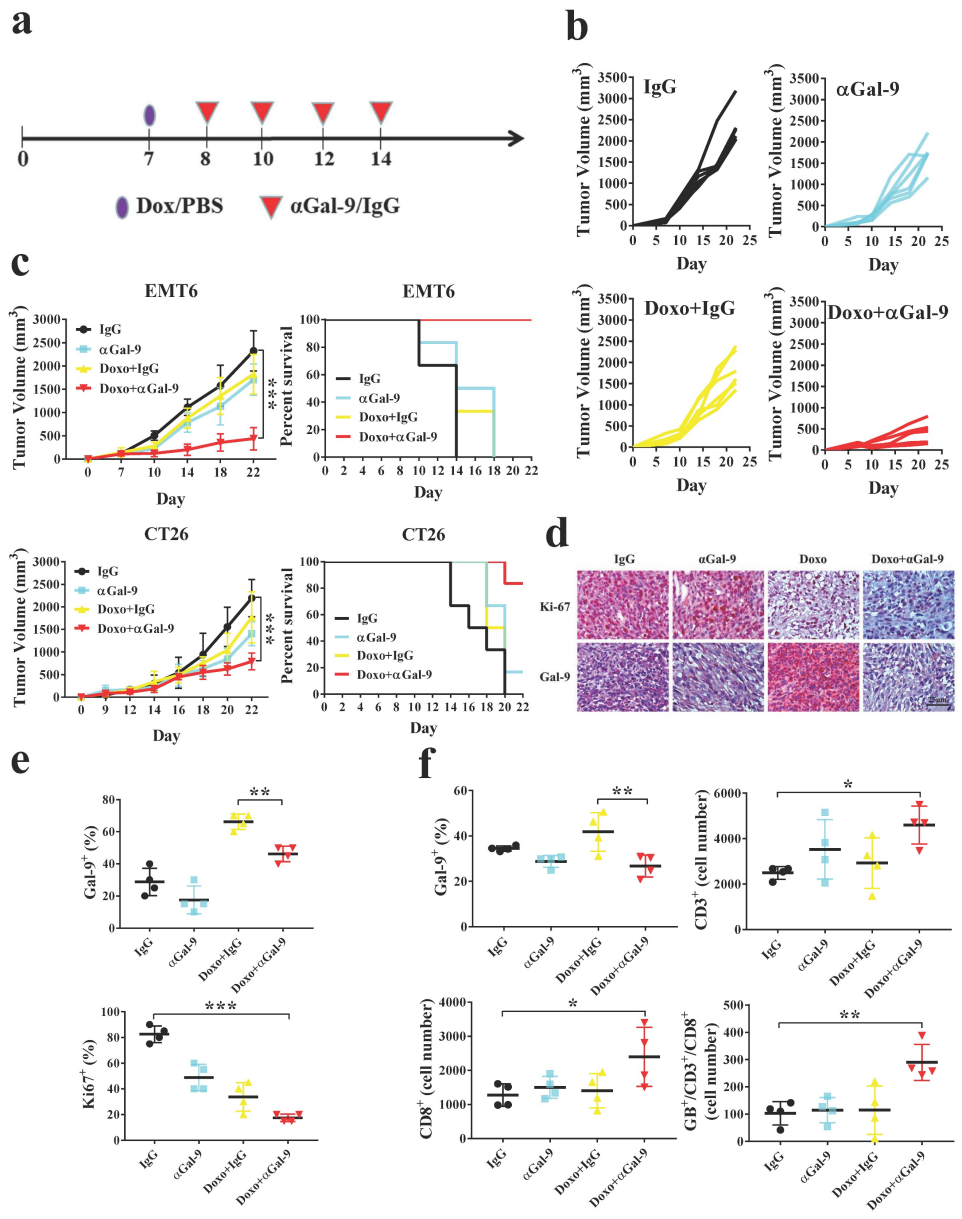
** Anti-Galectin-9 (αGal-9) therapy in combination with doxorubicin (Doxo) improves antitumor immunity and enhances cytotoxic T-cell activity.** (a) The experimental design on a syngeneic mouse model of subcutaneous EMT6 and CT26 cancer cell lines. Mice were randomly allocated into treatment with doxo (4 mg/kg) or PBS for 7-9 days. After indicated treatment, 100 μg of αGal-9 antibody or rat IgG were injected intraperitoneally every two days four times. Values represented mean ± SD of n=5 in each group. (b) Tumor volume was measured on the indicated different treatments and time points. (c) At indicated treatment, tumor volume and Kaplan-Meier survival curves were analyzed for mice bearing EMT6 or CT26 tumors. (d-e) Immunohistochemical staining of tumor sections revealed the expressions of Ki-67 (a proliferation marker) and Gal-9 under the indicated treatment in the tumor tissue. Scale bar, 50 µm. (f) The quantification of Gal-9^+^; CD3^+^; CD8^+^; granzymes b^+^ (GB^+^), and CD8^+^ plus CD3^+^ cell percentage in the tumor tissue from different groups of treatment.

To investigate whether Gal-9 blockade could enhance the antitumor efficacy of anthracyclines *in vivo*, we treated mice bearing EMT6 tumors with doxorubicin, Gal-9 mAb, doxorubicin plus Gal-9 mAb, or isotype control (Fig. 1A and Supplementary Fig. 1A). While doxorubicin or Gal-9 mAb alone showed a modest effect, their combined treatment demonstrated better efficacy in suppressing tumor growth and prolonging overall survival. (Fig. 1B, C and Supplementary Fig. 1B). Similar results were found in another syngeneic mouse model, CT26 colon cancer. In contrast, the combined treatment did not have an obvious effect on tumor growth in immune-deficient mice, suggesting that their efficacy relied on the competent immune system (Supplementary Fig. 1C). To validate our findings, we analyzed Gal-9 expression by performing IHC and found that Gal-9 levels were increased in tumor tissue isolated from mice treated with doxorubicin. The expression of nuclear protein Ki67 (pKi67), a tumor cell proliferation marker, was decreased in the combined treatment compared with each treatment alone (Fig. 1D, E and Supplementary Fig. 1D, E).

To investigate the mechanisms underlying the efficacy of the combined treatment, we harvested the tumors and analyzed the alteration of tumor-infiltrating lymphocytes (TILs) by flow cytometry. The results showed that doxorubicin significantly induces Gal-9 expression in EMT6 tumors *in vivo*. We found that the number of granzyme B-positive intratumoral CD8^+^ T cells was significantly increased in the combination treatment group compared with doxorubicin or anti-Gal-9 treatment alone (Fig. 1F). These results suggested that doxorubicin treatment may induce Gal-9 expression and that the combination of doxorubicin and anti-Gal-9 antibody enhances antitumor immunity in syngeneic mouse models.

### Anthracyclines upregulate Gal-9 expression in tumor cells

**Figure 2:**
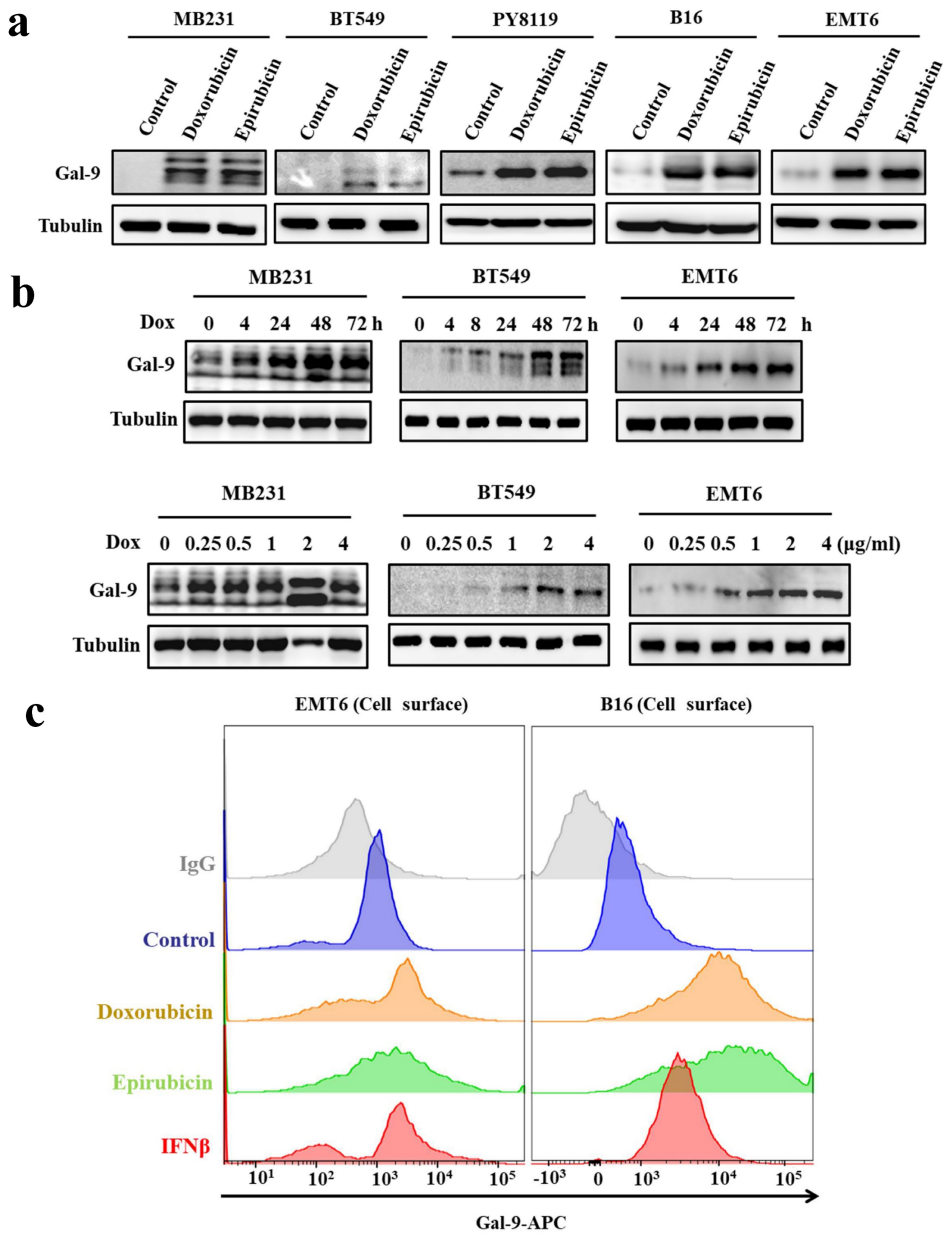
** Anthracyclines enhance Galectin-9 (Gal-9) expression in cancer cell lines.** (a) Western blot analysis for Gal-9 protein expression after treatment with 2 μg/ml of doxorubicin (Dox) or epirubicin in different cell lines (MB231, BT549, PY8119, B16, and EMT6) (b) Gal-9 protein expression after treatment with Dox with the indicated times or different concentrations (times: 0, 4, 24, 48 and 72 hours; different concentrations: 0, 0.125, 0.25, 0.5, 1 or 2 μg/ml) in different cell lines (MB231, BT549, and EMT6). Gal-9 protein levels were analyzed by Western blotting. (c) The cell surface Gal-9 expression was analyzed by flow cytometry in EMT6 and B16 cells. APC, allophycocyanin.

To further investigate the effect of anthracyclines on tumoral Gal-9 expression, we treated tumor cells with two anthracyclines, doxorubicin and epirubicin. The results showed that anthracycline treatment increased Gal-9 protein levels in all tested cell lines, including MDA-MB-231 and BT549 human breast cancer cells, PY8119 and EMT6 murine breast cancer cells, and B16-F10 murine melanoma cells (Fig. 2A). Furthermore, we found that doxorubicin induced Gal-9 expression in a time- and dose-dependent manner, as shown in Fig. 2B. Gal-9 expressed on the cell surface of cancer cells exerts immunosuppressive effects by binding to TIM-3 on T cells 12. Next, we examined whether the levels of cell surface Gal-9 were altered upon anthracycline treatment by flow cytometry analysis. As shown in Fig. 2C, anthracycline increased Gal-9 membrane levels in EMT6 and B16 murine cancer cells. Together, these results indicated that anthracyclines upregulate Gal-9 expression in cancer cells.

### Doxorubicin induces Galectin-9 expression via STING upregulation

**Figure 3:**
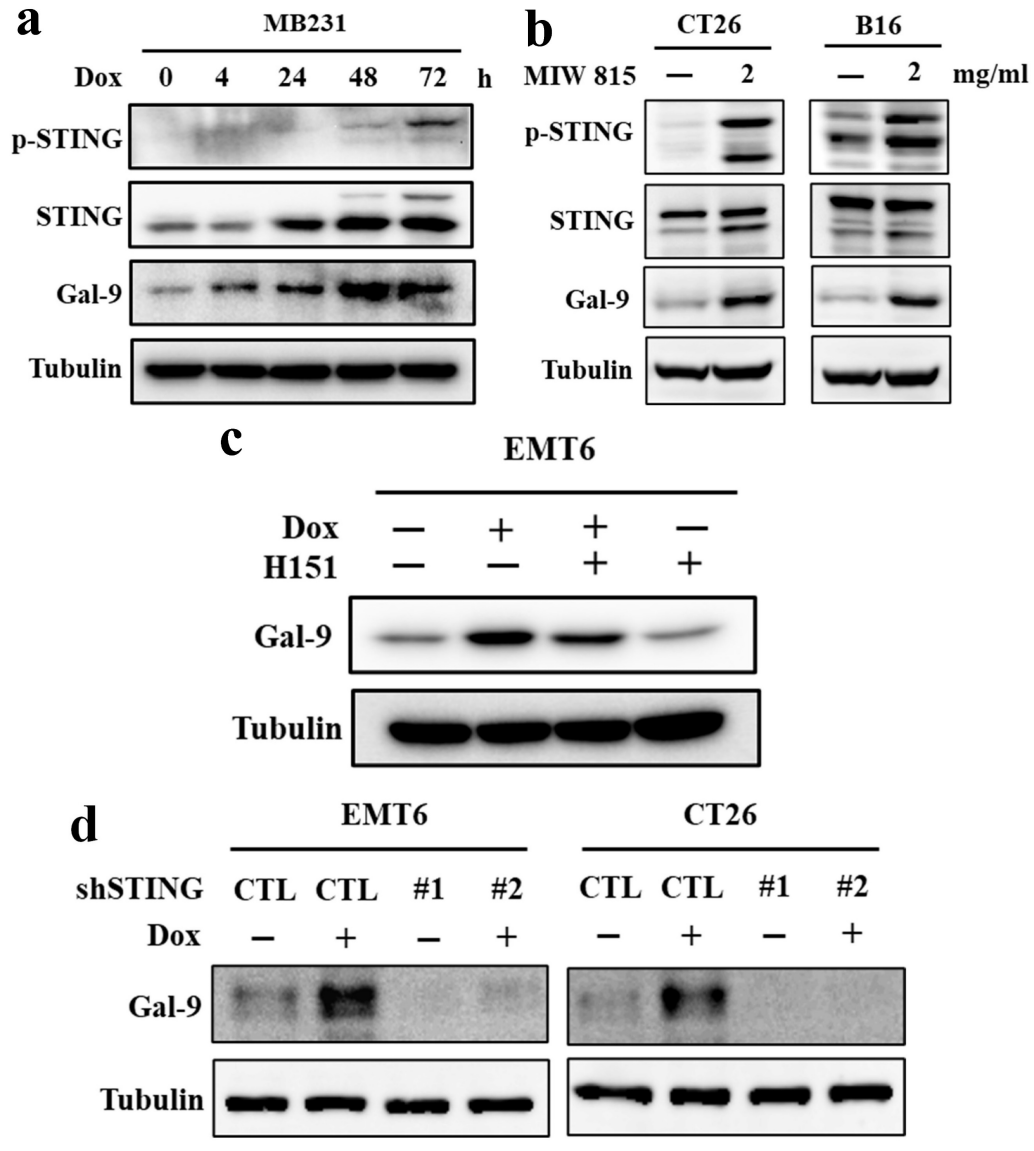
** Doxorubicin (Dox) induces Galectin-9 (Gal-9) expression through STING activation.** (a) MB231 cells were treated with Dox at the indicated times (hour) and subjected to immunoblotting with the indicated antibodies. (b) CT26 and B16 cells were treated with miw815 (2 mg/ml) for 24 hours. (c) EMT6 cells were treated with 2 μg/ml of doxorubicin and with or without 5 μM of STING inhibitor H151 for 24 hours. (d) EMT6 and CT26 parental or STING knockdown (KD) cells were treated with 2 μg/ml doxorubicin for 24 hours. The protein levels were analyzed by Western blotting.

In a recent study, the Gal-9 receptor TIM-3 was thought to inhibit the cGAS-STING pathway 18. Moreover, another study also showed that Gal-9 could induce STING degradation via TRIM29-mediated ubiquitination 19. Indeed, consistent with previous findings, phosphor-STING (p-STING) and STING levels were significantly increased by doxorubicin treatment in MD-MB-231 breast cancer cells (Fig. 3A). Furthermore, to identify the relationship between Gal-9 and STING in tumor cells upon doxorubicin treatment, we treated CT26 and B16 mouse cancer cell lines with the STING activator miw815. The results showed that the expression of Gal-9 and phospho-STING were both increased by treatment with doxorubicin and the STING activator miw815 (Fig. 3B). To test whether STING is involved in Gal-9 upregulation by doxorubicin, we treated the cells with H151, a STING inhibitor, and found that H151 significantly attenuated the increase in Gal-9 in response to doxorubicin (Fig. 3C). Furthermore, STING knockdown (KD) abrogated doxorubicin-induced Gal-9 in EMT6 and CT26 cells (Fig. 3D). Together, these results indicated that STING is required for doxorubicin-induced Gal-9 upregulation.

### IFNβ mediates doxorubicin-induced Gal-9 expression

**Figure 4:**
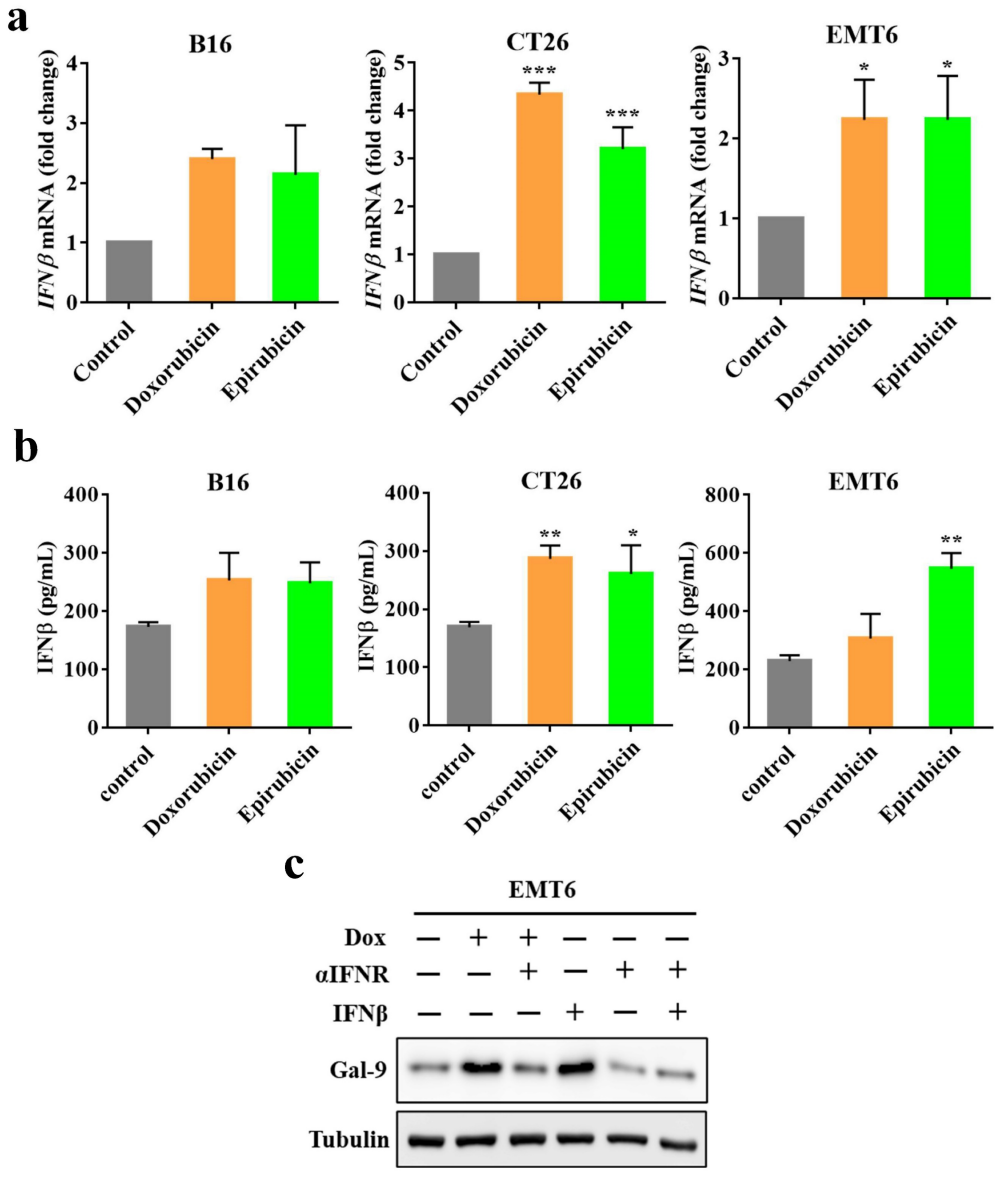
** Doxorubicin-induced Galectin-9 expression is highly correlated with IFNβ expression.** (a) The target genes (*IFNβ*) expression in different cell lines (B16, EMT6, and CT26) treated with doxorubicin or epirubicin for 24 hours were measured by Real-time PCR, normalized to GAPDH. (b) ELISA assay for Dox or epirubicin-induced IFNβ secretion in different cell lines (B16, EMT6, and CT26) in a conditioned medium. (c) Representative image of western blot analysis of Gal-9 in EMT6 cells after treatment with indicated doses of Dox, interferon receptor (IFNR) antibody, and IFNβ.

STING is critical for inducing IFN expression in many different types of cells 20. Moreover, we recently identified that IFNβ significantly influenced Gal-9 protein and mRNA expression 21. Therefore, to determine whether IFNβ is involved in anthracycline-induced Gal-9 expression, we first investigated IFNβ expression by real-time PCR in murine tumor cell lines (B16, EMT6, CT26) upon doxorubicin or epirubicin treatment. Indeed, doxorubicin or epirubicin treatment significantly induced IFNβ mRNA expression (Fig. 4A). We also examined the secretion levels of IFNβ in the conditioned medium in response to anthracyclines by ELISA. Compared with the control group, the level of IFNβ was increased after doxorubicin or epirubicin treatment (Fig. 4B). To examine whether IFNβ is required for Gal-9 induction in response to anthracyclines, we pretreated the cells with IFN receptor (IFNR) antibody to block the IFN-mediated pathway and found that the IFNR antibody significantly inhibited doxorubicin-induced Gal-9 protein expression (Fig. 4C). Together, these results suggested that the upregulation of Gal-9 expression by doxorubicin is dependent on IFNβ.

### Gal-9 levels are higher in the tumor tissues of chemotherapy-treated patients

**Figure 5:**
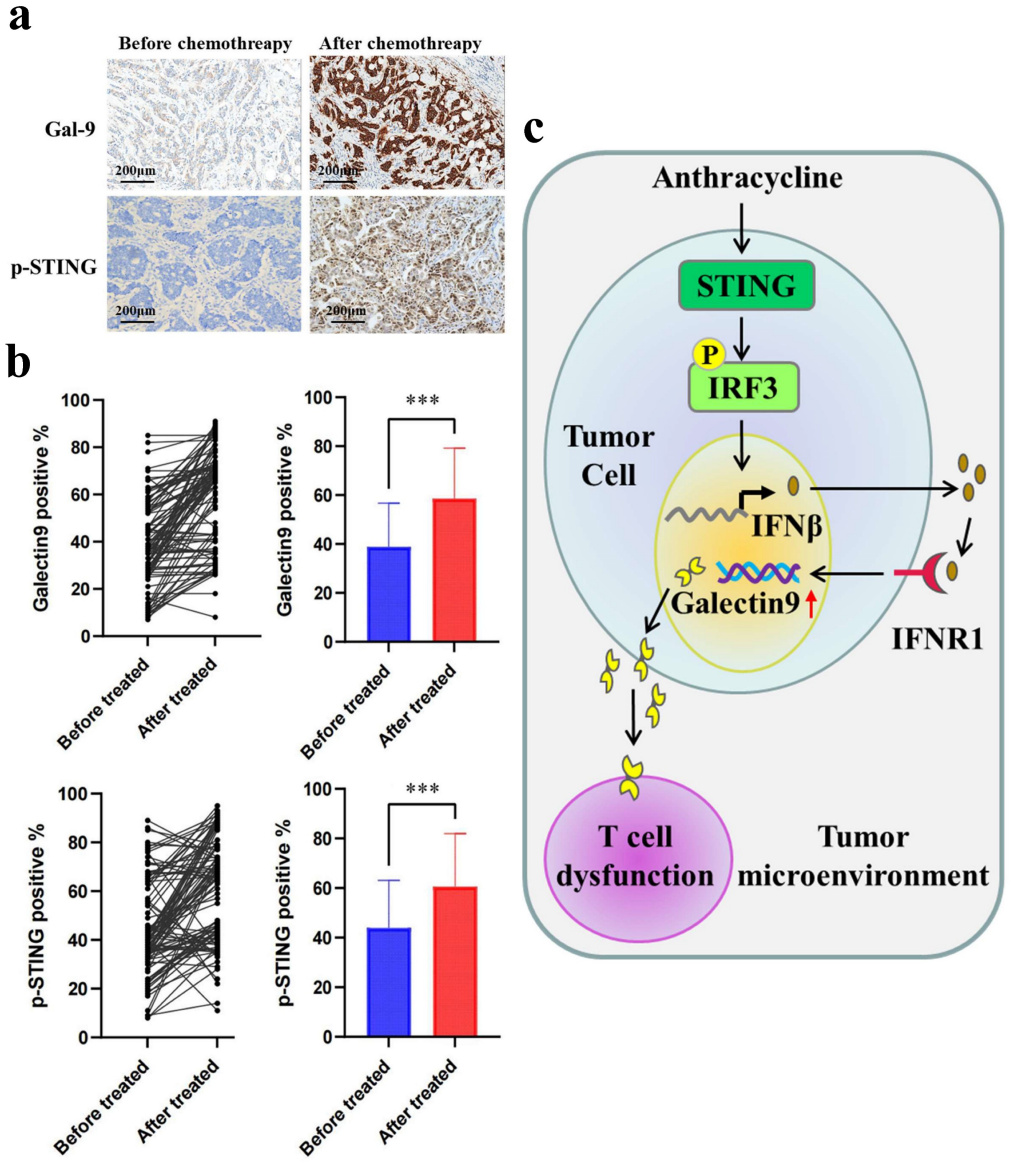
** Galectin-9 (Gal-9) and p-STING are upregulated in post-chemotherapy surgical breast cancer specimens.** (a) The images of IHC staining of p-STING and Gal-9 in human breast cancer tissues (n=104). Scale bar, 50 μm. (b) Gal-9 and p-STING staining in paired pre- and post-treatment of anthracycline samples show increased expression in post-treatment samples (P = 0.01). (c) A proposed model illustrating the mechanism of anthracyclines-induced immunosuppression is mediated by STING/Gal-9 axis in breast cancer.

Next, to evaluate the clinical relevance of our findings, we evaluated Gal-9 and p-STING levels by IHC in the tumor tissues of breast cancer patients before and after treatment with chemotherapy. Compared with the group before chemotherapy, higher levels of Gal-9 and p-STING were found in the patients posttreatment with anthracycline (Fig. 5A and 5B). Furthermore, Spearman's test showed a positive correlation between p-STING levels and Gal-9 expression (Table 1). Our results demonstrated STING/IFNβ-induced Gal-9 as a novel mechanism mediating the immunomodulation of chemotherapy. They suggested the combination of anthracycline and Gal-9 mAb as a promising therapeutic strategy for treating breast cancer (Fig. 5C).

## Discussion

In breast cancer, especially triple-negative breast cancer, chemotherapeutic agents such as anthracyclines are the most widely used and effective drugs. However, many patients are still resistant to anthracycline-based chemotherapy 22. Thus, improving breast cancer treatment regimens is still an urgent need. Moreover, a clinical study indicated that the level of TILs is critical for the effectiveness of anthracyclines 23. Therefore, enough activated TILs are essential to show the clinical benefit of anthracyclines in patients. In this study, we provided evidence that anthracyclines such as doxorubicin induce tumor Gal-9 expression via the STING-IFNβ axis. Gal-9 is known to inactivate CD8^+^ T cells. Thus, this pathway will limit the effectiveness of anthracyclines. Indeed, combining Gal-9 mAb with doxorubicin enhanced breast cancer treatment efficacy by improving CD8+ T-cell activity. In addition, we observed upregulated Gal-9 levels and p-STING levels in the tumor region after treatment with anthracyclines in chemotherapy-treated breast cancer patients. In summary, our findings resolved a molecular mechanism of anthracycline-induced STING that increases Gal-9 expression in cancer cells to inactivate T cells and suppress the effectiveness of chemotherapy. We further demonstrated that anti-Gal-9 mAb plus anthracyclines could be an effective therapeutic combination strategy for treating breast cancer. Thus, combined chemotherapy and anti-Gal-9, as reported in this study, are clinically important and worthy of further testing in the clinic.

Doxorubicin has been one of the most effective anticancer drugs against solid tumors of diverse origins for many years 24. The main mechanism of doxorubicin is insertion into DNA and inhibition of macromolecular biosynthesis 25. In addition, recent studies have demonstrated that doxorubicin can contribute to reestablishing antitumor immunity through ICD regulation 26. However, how ICD constructs an immunosuppressive cancer microenvironment is still largely unknown. ICD is a phenomenon that can change the composition of the cell surface and release cell soluble mediators such as ATP and high mobility group Box 1 (HMGB1), which are passively released by cancer cells undergoing ICD, triggering immune cell maturation 27. However, cancer cells have also been shown to inhibit the expression of HMGB1, consequently decreasing immune infiltration in several cancers 28. In addition, another study showed that cancer cell-secreted gelsolin (GSN) disrupts immunosurveillance by competitively blocking the interaction between extracellular F-actin and DNGR-1 on dendritic cells under ICD stimulation 29. Moreover, we showed that doxorubicin induces Gal-9 expression and that blockade of Gal-9 by an antibody enhances antitumor immunity by activating T cells. These data suggested that targeting Gal-9 might be an attractive immunotherapy strategy for breast cancer. These results reflected the mechanisms by which malignant cells evade ICD-driven immunity and highlighted the critical relevance of these pathways for immunosurveillance in breast cancer.

The cyclic GMP-AMP synthase-stimulator of interferon genes (cGAS) and stimulator of interferon response cGAMP interactor (STING) pathways have been demonstrated to be key in detecting intracellular DNA. This pathway links DNA sensing with a robust innate immune defense program 30. The mechanism is activated upon binding to double-stranded DNA (dsDNA) and activating STING as an adapter protein on the endoplasmic reticulum (ER) membrane. Upon triggering a signaling cascade, this pathway produces a series of immune and inflammatory mediators, which are important products in inflammatory and tumor biology 31. Recent studies have demonstrated that several conditions in cancer cells contribute to activating the cGAS-STING pathway, such as chromosome mis-segregation during cell division or mitochondrial DNA leakage under the reactive oxygen species (ROS) response 32. In addition, another study indicated that inhibition of the DNA repair-related gene ATM activates the cGAS/STING pathway by downregulating mitochondrial transcription Factor A (TFAM) 33. Our study discovered that doxorubicin induces STING expression in breast cancer cell lines. Clinically, we further showed that breast cancer patients, after treatment with chemotherapy, had significantly enhanced p-STING and Gal-9 expression in tumor tissues, which explains the regulation of ICD-induced tumor immunosuppression in the tumor microenvironment. In addition, previous studies have also shown that SHP2 induces cGAS-STING activation by promoting DNA damage and leads to the promotion of IFN expression by STING, which in turn supports a positive feedback loop to enhance RNA and DNA expression, including inflammation, senescence, and autophagy 34. Moreover, clinically, another report revealed that after neoadjuvant chemotherapy, the cGAS-STING-activated immune response could be a biomarker to predict the effect of chemotherapy 35. Recent studies have also demonstrated that STING can be phosphorylated by upstream signaling, such as EGFR or other activators, to induce innate immunity in cells 36. Furthermore, STING phosphorylation is important for inducing downstream IRF3 phosphorylation 37. All these findings suggest that STING is a potential target for combination with anthracyclines to treat breast cancer.

Gal-9, an important family member, consists of two carbohydrate-recognition domains (CRDs) connected by a linker sequence 38. This structure can help Gal-9 crosslink glycoproteins and then form multivalent galectin-glycoprotein lattices that regulate multiple biological functions and exert strong immunomodulatory effects, including mediating T-cell responses, cell adhesion, cell surface recognition, migration, and chemoattraction and acting as a modulator of important signals between growth and apoptosis 39. In addition, other studies have demonstrated that Gal-9 plays an important role in antitumor immunity and is involved in tumor progression, including tumor cell adhesion, survival, immune escape, and angiogenesis 40. However, Gal-9 has also previously been shown to bind 4-1BB to facilitate the functional activities of lymphocytes 14. However, our recent study found that Gal-9 induces T-cell death by interacting with PD-1 and TIM-3 to induce apoptosis 21. Moreover, another group also demonstrated that Gal-9 exerts critical immune-suppressive effects specific to dectin 1 signaling 13. These studies suggest that Gal-9 is a potential prognostic biomarker and a promising treatment target for certain malignancies. However, the potential mechanism of Gal‐9 involvement in breast cancer is still uncertain. IFN-γ or IL-1β can induce gal-9 expression in tissues such as endothelial cells, fibroblasts, and astrocytes 41. Moreover, another study demonstrated that obesity induces Gal-9 upregulation in B-acute lymphoblastic leukemia cells by the adipocyte secretome 21. The current study revealed that IFNβ induces Gal-9 expression via STING activation in breast cancer cell lines. Interestingly, a recent study indicated that Gal-9 promotes immunosuppression in the tumor microenvironment by inducing STING degradation 19. This finding arouses our interest in STING-Gal-9 as a potential negative feedback regulator that causes decreased TIL activity.

In summary, our data revealed that anthracycline treatment induced tumor Gal-9 expression by inducing STING-mediated IFNβ. Furthermore, we also showed Gal-9 upregulation in the tumor tissues of breast cancer patients after anthracycline treatment. Preclinically, in our mouse model, inhibition of tumor Gal-9 by mAB treatment subsequently activates CTL activity, which promotes antitumor immunity. Moreover, combination treatment with anthracyclines and Gal-9 mAB significantly reduced tumor burden and OS in breast cancer immune-competent mouse models. This study provides a scientific basis to develop a novel effective combination therapy for breast cancer.

## Supplementary Material

Supplementary figure.

## Figures and Tables

**Table 1:**
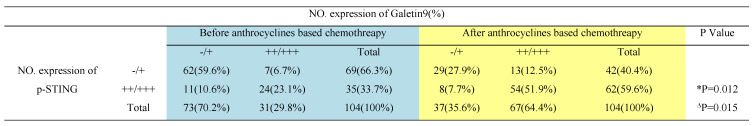
Correlation between p-STING and Galectin-9 (Gal-9) in before or after chemotherapy surgical breast cancer specimens.

## Abbreviations

ICD: Immunogenic cell death
DAMPs: damage-associated molecular patterns
CRT: calreticulin
IFNs: Interferons
PRR: Pattern recognition receptor
TLR3: Toll-like receptor 3
Gal-9: Galectin-9
FBS: fetal bovine serum
RT-PCR: Real-time PCR
mAb: monoclonal antibody
PBS: phosphate-buffered saline
CTL: cytotoxic T lymphocytes
TILs: tumor-infiltrating lymphocytes
KD: knockdown
IFNR: IFN receptor
HMGB1: high mobility group box 1
GSN: gelsolin
cGAS: Cyclic GMP-AMP synthase-stimulator of interferon genes
STING: stimulator of interferon response cGAMP interactor
dsDNA: double-stranded DNA
ER: endoplasmic reticulum
ROS: reactive oxygen species
TFAM: transcription factor A
CRDs: carbohydrate-recognition domains
