# Supplementary material for: Inhibition of Galectin-9 sensitizes tumors to anthracycline treatment via inducing antitumor immunity
Source: Int J Biol Sci. 2023 Aug 28;19(14):4644–56. doi: 10.7150/ijbs.84108 (PMC10535704; doi:10.7150/ijbs.84108)
Supplement: Supplementary file 1 — Supplementary figure. [file ijbsv19p4644s1.pdf]

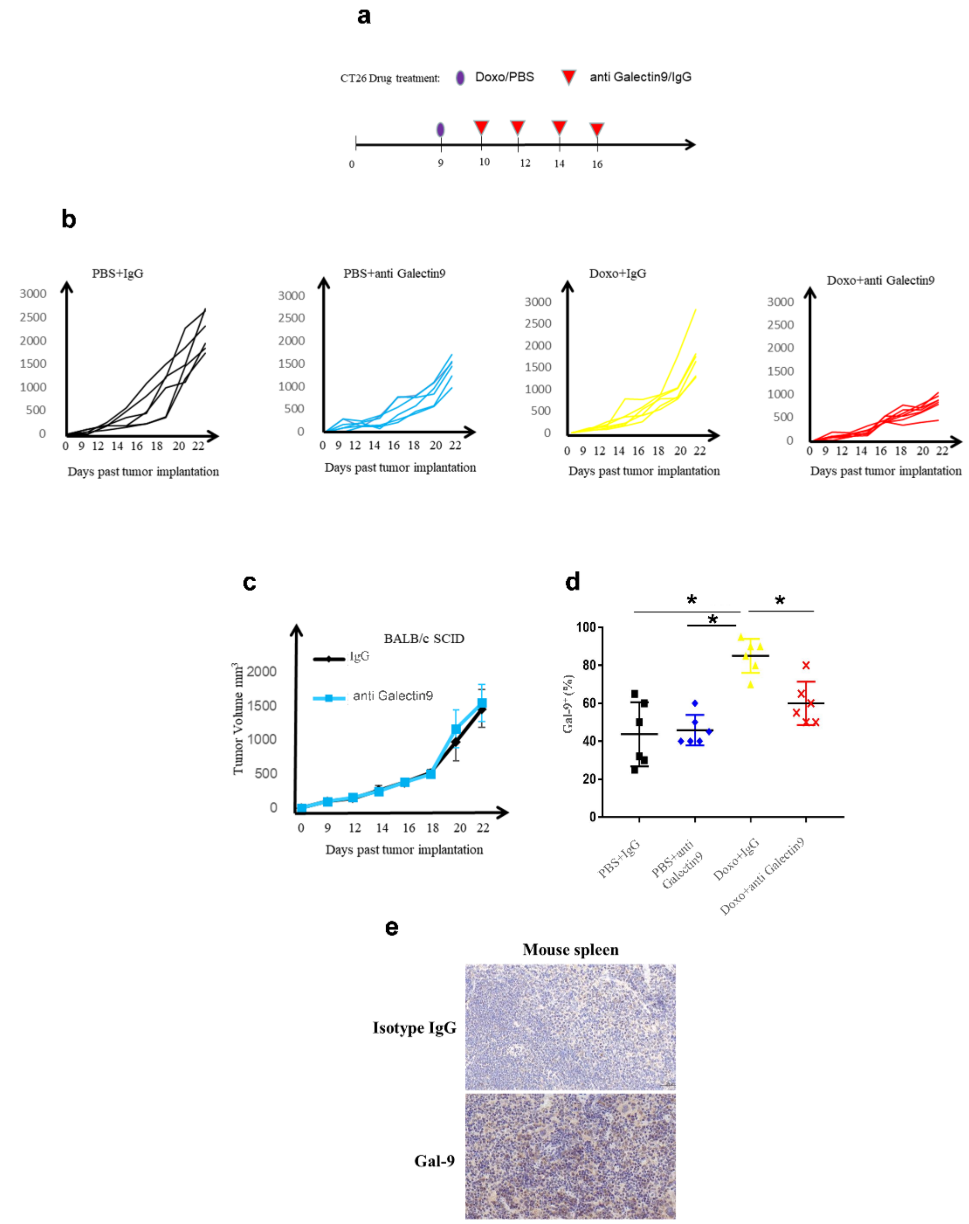

Supplementary Fig 1. Anti-Galectin-9 (αGal-9) therapy in combination with doxorubicin enhances antitumor activity in CT26 syngeneic mouse model.

(a) The experimental design of CT26 animal model. Mice were randomly treated with doxorubicin (Doxo) (4 mg/kg) or PBS for 9 days (day 9). After

indicated treatment, 100 μg of αGal-9 antibody or anti-IgG were injected intraperitoneally every 2 days for 4 times. (b) Tumor volume was measured on different treatments (PBS+IgG, PBS+αGal-9, Doxo+IgG, Doxo+ αGal-9). Values represented mean ± SD of n=5 in each group. (c) The experimental design used subcutaneous of EMT6 and CT26 cancer cell lines in immunodeficient mice (SCID). Mice were randomly treated with IgG or αGal-9. Tumor volume was measured on different treatments. Values represented mean ± SD of n=5 in each group. (d) The quantification of immunohistochemical staining of Gal-9 percentage in the tumor tissue from different groups of treatment. (e) The immunohistochemical staining of isotype IgG and Gal-9 antibody percentage in the mouse spleen tissue.
